# Induced Systemic Resistance (ISR) and Fe Deficiency Responses in Dicot Plants

**DOI:** 10.3389/fpls.2019.00287

**Published:** 2019-03-11

**Authors:** Francisco J. Romera, María J. García, Carlos Lucena, Ainhoa Martínez-Medina, Miguel A. Aparicio, José Ramos, Esteban Alcántara, Macarena Angulo, Rafael Pérez-Vicente

**Affiliations:** ^1^Department of Agronomy, Campus de Excelencia Internacional Agroalimentario CeiA3, Universidad de Córdoba, Córdoba, Spain; ^2^Department of Botany, Ecology and Plant Physiology, Campus de Excelencia Internacional Agroalimentario CeiA3, Universidad de Córdoba, Córdoba, Spain; ^3^Molecular Interaction Ecology, German Centre for Integrative Biodiversity Research (iDiv) Halle-Jena-Leipzig, Leipzig, Germany; ^4^Department of Microbiology, Campus de Excelencia Internacional Agroalimentario CeiA3, Universidad de Córdoba, Córdoba, Spain

**Keywords:** dicotyledons, ethylene, iron, ISR, rhizobacteria, rhizofungi, rhizosphere, stress responses

## Abstract

Plants develop responses to abiotic stresses, like Fe deficiency. Similarly, plants also develop responses to cope with biotic stresses provoked by biological agents, like pathogens and insects. Some of these responses are limited to the infested damaged organ, but other responses systemically spread far from the infested organ and affect the whole plant. These latter responses include the Systemic Acquired Resistance (SAR) and the Induced Systemic Resistance (ISR). SAR is induced by pathogens and insects while ISR is mediated by beneficial microbes living in the rhizosphere, like bacteria and fungi. These root-associated mutualistic microbes, besides impacting on plant nutrition and growth, can further boost plant defenses, rendering the entire plant more resistant to pathogens and pests. In the last years, it has been found that ISR-eliciting microbes can induce both physiological and morphological responses to Fe deficiency in dicot plants. These results suggest that the regulation of both ISR and Fe deficiency responses overlap, at least partially. Indeed, several hormones and signaling molecules, like ethylene (ET), auxin, and nitric oxide (NO), and the transcription factor MYB72, emerged as key regulators of both processes. This convergence between ISR and Fe deficiency responses opens the way to the use of ISR-eliciting microbes as Fe biofertilizers as well as biopesticides. This review summarizes the progress in the understanding of the molecular overlap in the regulation of ISR and Fe deficiency responses in dicot plants. Root-associated mutualistic microbes, rhizobacteria and rhizofungi species, known for their ability to induce morphological and/or physiological responses to Fe deficiency in dicot plant species are also reviewed herein.

## Introduction

In the last decades, crop productivity has been mainly based on the use of high-yielding varieties and in the application of high amounts of fertilizers and pesticides. Despite crop protection measures, current losses are estimated at 20–40% for the major food crops world-wide (Savary et al., 2012). Hence, novel strategies for crop production, with less reliance on chemical products need to be developed. In relation to plant mineral nutrition, two strategies that can contribute to this goal are the development of crop varieties more efficient in nutrient acquisition and better management of the rhizosphere (Shen et al., 2013). The rhizosphere, the soil volume influenced by the root system, is one of the most energy-rich habitats on Earth, allowing the life of a myriad of microbes (Pieterse et al., 2014; Pii et al., 2015). Many of them are pathogenic and threaten plant growth. However, there are also many others that are beneficial for plants, like rhizobacteria (“PGPB or PGPR: Plant Growth-Promoting Bacteria or Rhizobacteria”) and fungi (“PGPF: Plant Growth-Promoting Fungi”), which can improve plant growth and benefit the adaptation of plants to adverse conditions (Yang et al., 2008; de Zelicourt et al., 2013; Pieterse et al., 2014; Pii et al., 2015; Verbon and Liberman, 2016). Some rhizosphere microbes can have negative effects on plant mineral nutrition, for example, by competing with plants for some nutrients. However, several genera of the rhizosphere microbiota can facilitate nutrient acquisition by plants, thus having positive effects. These beneficial microbes include, among others, mycorrhizal fungi and *Rhizobium*, which establish mutualistic symbiosis with plant roots that improve phosphorus (P) or nitrogen (N) nutrition, respectively (Guinel, 2015; Wang W. et al., 2017). Additionally, there are free-living mutualistic microbes that can improve plant nutrition through different mechanisms, such as the release of nutrient solubilizing compounds or the modification of root physiology and architecture (Jin et al., 2014; Mimmo et al., 2014; Zamioudis et al., 2014, 2015; Contreras-Cornejo et al., 2015; Li et al., 2015; Pii et al., 2015; García-López et al., 2016; Garnica-Vergara et al., 2016; Verbon et al., 2017).

Among the essential mineral nutrients required by plants, iron (Fe), along with P and N, represent the major constraints for crop productivity worldwide (Pii et al., 2015; Scagliola et al., 2016; Tsai and Schmidt, 2017b). Iron deficiency is widely distributed, mainly in calcareous soils (approximately one third of cultivated lands) which are abundant in arid and semiarid regions (Briat et al., 2015). To cope with Fe deficiency, plants develop morphological and physiological responses, mainly in their roots, aimed to facilitate its acquisition (see following Section; Kobayashi and Nishizawa, 2012; Brumbarova et al., 2015; Lucena et al., 2015). Despite these responses, in many cases it is necessary to apply Fe fertilizers to correct Fe deficiency. For Fe supply in the field, the most common practice is the application of Fe chelates to soils, which are generally expensive and therefore restricted to high added-value field-grown crops (Briat et al., 2015). An alternative is the use of more Fe efficient plant genotypes. However, different results obtained with sterile soils have shown that, even with these genotypes, the cooperation of rhizosphere microbes is necessary for an adequate Fe acquisition (Jin et al., 2014; Pii et al., 2015).

Several studies demonstrated that the application of some beneficial microbes to soils can improve the Fe nutrition of plants (de Santiago et al., 2009, 2013; Zhang et al., 2009; Freitas et al., 2015; Li et al., 2015; Ipek et al., 2017; Sonbarse et al., 2017; Aras et al., 2018; Arıkan et al., 2018). However, the main mechanisms driving such effects are complex and not fully understood. One possible mechanism is the release of Fe solubilizing compounds to soils (Jin et al., 2014; Mimmo et al., 2014; Pii et al., 2015). Moreover, the rhizosphere mutualistic microbiota can also improve plant Fe uptake by the alteration of the root physiology and architecture (Zamioudis et al., 2014, 2015; Contreras-Cornejo et al., 2015; Garnica-Vergara et al., 2016; Scagliola et al., 2016; Verbon et al., 2017). In the last years it has been found that some rhizosphere microbes can induce physiological and morphological responses in roots of dicot plants similar to the ones induced by plants under Fe deficiency (Zhang et al., 2009; Orozco-Mosqueda et al., 2013; Jin et al., 2014; Pieterse et al., 2014; Zamioudis et al., 2014, 2015; Zhao et al., 2014; Pii et al., 2016b; Zhou et al., 2016a; Martínez-Medina et al., 2017; Verbon et al., 2017). It is remarkable that these rhizosphere microbes are also capable of eliciting the Induced Systemic Resistance (ISR) against pathogens and insects. This observation suggests that both processes (ISR and Fe deficiency responses) might be closely interconnected, and opens new possibilities for optimizing the management of the rhizosphere microbiota for improving Fe nutrition and health (Pieterse et al., 2014; Zamioudis et al., 2014, 2015; Verbon et al., 2017). However, the nodes of convergence between the two processes remain unclear.

Elucidating the main nodes of interconnection between the pathways regulating microbe-elicited ISR and Fe uptake is critical for optimizing the use of plant mutualistic microbes in agriculture. This review summarizes the progress in the understanding of the molecular overlap in the regulation of ISR and Fe deficiency responses in dicot plants. We further describe and evaluate rhizobacteria and rhizofungi species, known for their ability to induce morphological and/or physiological responses to Fe deficiency in dicot plants and with potential for a future use as Fe biofertilizers.

## Fe Deficiency Responses in Dicot Plants

Iron (Fe) is abundant in most soils, mainly as Fe^3+^, although its availability to plants is low, especially in calcareous soils (Briat et al., 2015). Based on the mechanisms used by plant roots to facilitate mobilization and uptake of Fe, plants are classified into Strategy I species (dicots and non-grass monocots) and Strategy II species (grasses; Kobayashi and Nishizawa, 2012; Ivanov et al., 2012). Dicots, such as *Arabidopsis* and tomato, are Strategy I species which have to reduce Fe^3+^ to Fe^2+^ at the root surface, by means of a ferric reductase (encoded by *FRO2* in *Arabidopsis*), prior to its subsequent uptake through a Fe^2+^ transporter (encoded by *IRT1* in Arabidopsis; Ivanov et al., 2012; Kobayashi and Nishizawa, 2012). This review is devoted to dicots, where ISR mechanisms have been more extensively studied (Balmer et al., 2013). Consequently, the mechanisms described thereafter correspond to Strategy I plant species. For details about the Strategy II plant species readers are referred to other articles in this special issue.

When grown under Fe deficiency, Strategy I species develop several physiological and morphological responses, mainly in roots, known as Fe deficiency responses. Those responses are aimed at facilitating Fe mobilization and uptake (Ivanov et al., 2012; Kobayashi and Nishizawa, 2012; Brumbarova et al., 2015; Lucena et al., 2015). Among the physiological responses are: an enhanced ferric reductase activity due to upregulation of the *FRO* genes; an enhanced Fe^2+^ uptake capacity due to upregulation of the *IRT1* genes; the acidification of the rhizosphere due to upregulation of *AHA* or *HA* (H^+^-ATPase) genes (Waters et al., 2007; Brumbarova et al., 2015; Lucena et al., 2015); an increase of the synthesis and release of organic acids, like citrate and malate, to the medium (Kabir et al., 2012; Schmidt et al., 2014); an increase of the synthesis and release of phenolic compounds to the medium due to upregulation of genes like *F6′H1*, *S8H*, *BGLU42*, and *ABCG37* (Schmid et al., 2014; Schmidt et al., 2014; Zamioudis et al., 2014; Tsai and Schmidt, 2017a; Siwinska et al., 2018; Tsai et al., 2018); and an increase of the synthesis and release of flavins to the medium (Rodríguez-Celma and Schmidt, 2013). The acidification facilitates the solubilisation of Fe and the functioning of the ferric reductase which has an optimum pH around 5.0 (Lucena et al., 2007; Waters et al., 2007). Organic acids can act as chelating agents for Fe in the soil and also inside the plant (Durrett et al., 2007; Schmidt et al., 2014). In fact, Fe is moved through the xylem chelated with citrate (Durrett et al., 2007; Schmidt et al., 2014). Phenolic compounds, like coumarins, and flavins can act as chelating and reducing agents of Fe^3+^, thus facilitating its mobilization in the rhizosphere (Rodríguez-Celma and Schmidt, 2013; Tsai and Schmidt, 2017a; Rajniak et al., 2018). The *F6′H1* (“Feruloyl-CoA 6′-Hydroxylase1”) and *S8H* (“Scopoletin 8-Hydroxylase”) genes encode enzymes involved in the last steps of the synthesis of the coumarins scopoletin and fraxetin (Schmid et al., 2014; Schmidt et al., 2014; Tsai and Schmidt, 2017a; Siwinska et al., 2018; Tsai et al., 2018). The *ABCG37* gene (also named *PDR9*) encodes an ABC transporter involved in the release of coumarins to the medium (Fourcroy et al., 2014, 2016; Zamioudis et al., 2014) while the *BGLU42* gene encodes a β-glucosidase, possibly required for the processing of glycosylated phenolic compounds as an essential step for their secretion in the root vicinity (Zamioudis et al., 2014; Stringlis et al., 2018b). Among the morphological responses are: development of subapical root hairs, cluster roots, and transfer cells, all of which are aimed to increase the surface of contact with the soil (Römheld and Marschner, 1986; Lucena et al., 2015; Romera et al., 2017). Both physiological and morphological responses are mainly located in the subapical regions of the roots (Römheld and Marschner, 1986).

The regulation of the physiological and morphological responses described above is not fully understood but in the last years several transcription factors (TFs) that participate in the activation of most of their associated genes have been described (Ivanov et al., 2012; Kobayashi and Nishizawa, 2012; Brumbarova et al., 2015; Zhang et al., 2015; Li et al., 2016; Liang et al., 2017). In *Arabidopsis*, the master regulator of most of these genes is FIT (bHLH29), homolog of the tomato FER (Bauer et al., 2007 and references therein). The FIT regulatory network comprises other bHLH TFs of the Ib subgroup, such as bHLH38, bHLH39, bHLH100, and bHLH101. All of them have redundant functions and can interact with FIT to form heterodimers that activate the expression of the Fe acquisition genes *FRO2* and *IRT1* (Yuan et al., 2008; Wang N. et al., 2013; Brumbarova et al., 2015). *FIT/FER* is induced in roots in response to Fe deficiency while the other Ib bHLH genes cited above are induced in both roots and leaves in response to Fe deficiency (Brumbarova et al., 2015 and references therein). FIT also controls MYB10 and MYB72, two other TFs essential for plant growth on low Fe conditions (Palmer et al., 2013; Zamioudis et al., 2014, 2015). Besides the FIT/Ib bHLH regulatory network, there is another regulatory network related to the POPEYE (PYE; bHLH47) TF and associated with the vasculature (Brumbarova et al., 2015). In the last years, it has been found that, under Fe-deficiency conditions, IVc subgroup bHLH TFs [bHLH34, bHLH104, bHLH105(ILR3), and bHLH115] activate *FIT/bHLH38/39/100/101* and *PYE* expression (Zhang et al., 2015; Li et al., 2016; Liang et al., 2017). Upstream of the IVc subgroup bHLH TFs is the BRUTUS (BTS) protein, which possesses Fe-binding domains and that interacts with IVc bHLH TFs, targeting them for proteasomal degradation (Zhang et al., 2015; Liang et al., 2017). Since the IVc bHLH TFs act as positive regulators of Fe deficiency responses, the current data suggests that BTS is a negative regulator of Fe deficiency responses (Zhang et al., 2015; Hindt et al., 2017).

**FIGURE 1:**
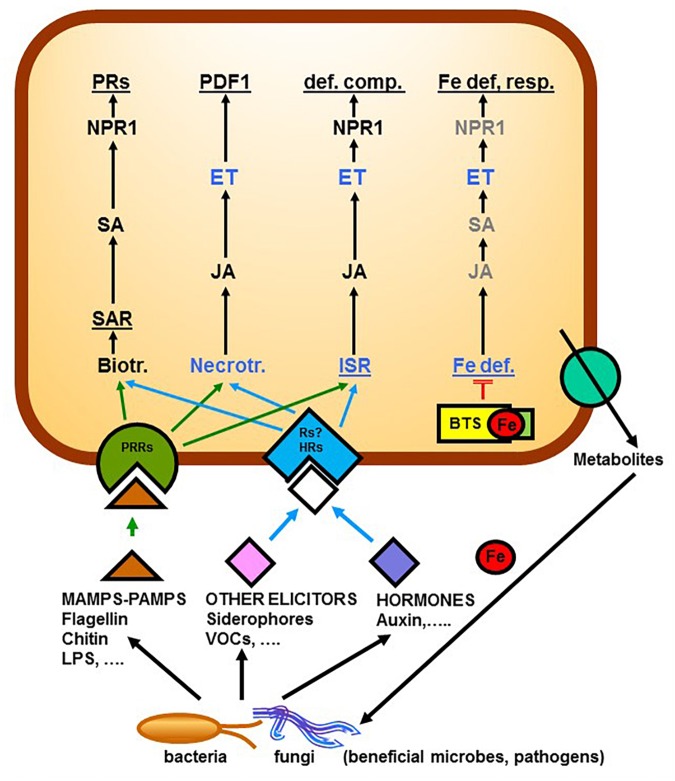
Relationship between Fe deficiency responses in dicots and responses to pathogens (necrotrophs or biotrophs that elicit SAR) or to beneficial microbes that elicit ISR. Microbes can produce Microbe-Associated Molecular Patterns (MAMPs) or Pathogen-Associated Molecular Patterns (PAMPs), like flagellin, that are perceived by PRRs, or other elicitors, like Volatile Organic Compounds (VOCs), or siderophores, that are perceived by Rs (some of them unknown). In some cases, microbes themselves can produce hormones, like auxin, that are perceived by HRs. After the perception of the eliciting or hormonal signals by receptors, there is activation of signaling pathways including hormones that lead to the activation of the different responses. In the case of Fe, BTS binds Fe (probably a chelated form of Fe) and, in this way, blocks the signaling pathway leading to the activation of Fe deficiency responses. In several of these pathways (responses to necrotrophs, ISR and Fe deficiency) ET plays a key role. Roots can also release different metabolites, like coumarins, that can shape the rhizosphere microbiome. In gray are components whose participation in the process is not yet clear. BTS (Brutus protein), def. comp. (defensive compounds), ET (Ethylene), Fe def. resp. (Fe deficiency responses), HRs (Hormonal Receptors), ISR (Induced Systemic Resistance), JA (Jasmonic Acid), LPS (Lipopolysaccharides), MAMPs (Microbe-Associated Molecular Patterns), NPR1 (Nonexpressor of PR genes1), PAMPs (Pathogen-Associated Molecular Patterns), PDF1 (Plant Defensin), PRs (Pathogenic-Related proteins), PRRs (Pattern Recognition Receptors), SA (Salicylic Acid), Rs (Receptors), SAR (Systemic Acquired Resistance), VOCs (Volatile Organic Compounds). Based on Pieterse et al., 1998, 2012; Lucena et al., 2015; García et al., 2018; Stringlis et al., 2018b; Tyagi et al., 2018.

The mechanisms by which plants perceive Fe deficiency and how this perception is transmitted to the activation of the responses is not fully understood. Several studies support a role for hormones and other plant signaling molecules in the activation of FIT and other TFs and, consequently, in the upregulation of the ferric reductase, the Fe^2+^ transporter and other Fe-related genes. Within them, the plant hormone ethylene (ET) has been found to play a key role in the regulation of most of the physiological and morphological responses to Fe deficiency (Figure 1; reviewed in Lucena et al., 2015; Li and Lan, 2017; Romera et al., 2017). Besides ET, auxin, nitric oxide (NO), sucrose, and glutathione (GSH) have also been involved in the regulation of Fe deficiency responses; all of them increase in Fe-deficient roots although their specific roles are not fully understood (Romera et al., 1999, 2011, 2017; Lucena et al., 2006, 2015; Graziano and Lamattina, 2007; Waters et al., 2007; Bacaicoa et al., 2009, 2011; García et al., 2010, 2011; Chen et al., 2010; Lingam et al., 2011; Meiser et al., 2011; Koen et al., 2012; Yang et al., 2014; Shanmugam et al., 2015; Lin et al., 2016; Li and Lan, 2017; Kailasam et al., 2018). By contrast to these activating signals, other ones have been implicated in the suppression of Fe deficiency responses, like cytokinins (Séguéla et al., 2008), jasmonic acid (JA; Maurer et al., 2011), brassinosteroids (Wang et al., 2012), and some phloem Fe-related signals (García et al., 2013, 2018). To integrate both positive and negative signals in the regulation of Fe acquisition genes in roots, a model has been proposed where auxin/ET/NO would act as activators of their expression, while LODIS (“LOng Distance Iron Signal”: a phloem Fe-related signal) would act to repress them (Lucena et al., 2006; García et al., 2011, 2018; Romera et al., 2011, 2017).

## Induced Systemic Resistance (ISR)

Besides responses to abiotic stresses, plants also respond to biotic stresses provoked by biological agents, like pathogens or insects (Pieterse et al., 2014; Martínez-Medina et al., 2017; Verbon et al., 2017). Some of these responses are localized but others are systemic, spreading far from the attacked organ and inducing defensive responses in the entire plant (Pieterse et al., 2014; Verbon et al., 2017). Within this second possibility, induced resistance is a physiological state of enhanced defensive capacity of the plant triggered by biological or chemical inducers, which protects plant tissues not exposed to the initial attack against future attack by pathogens and herbivorous insects (Van Loon et al., 1998). Induced resistance can be triggered in plants by the infection of pathogens, in response to insect herbivory, or upon root colonization by certain rhizosphere mutualistic microbes. Two of the most studied forms of induced resistance are SAR (Systemic Acquired Resistance), triggered by plant pathogens, and ISR, triggered by root-colonizing mutualistic microbes, like *Pseudomonas simiae* (syn. *Pseudomonas fluorescens*), *Paenibacillus polymyxa*, or *Trichoderma* spp. (Table 1; Zhang et al., 2009; Pieterse et al., 2012, 2014; Alizadeh et al., 2013; Zamioudis et al., 2014, 2015; Zhao et al., 2014; Martínez-Medina et al., 2017; Verbon et al., 2017). SAR and ISR are mainly differentiated on the basis of the elicitor and the regulatory pathways involved, though the signaling pathways that regulate SAR and ISR share some components (Pieterse et al., 1998, 2002, 2012, 2014; Van Loon et al., 1998; Choudhary et al., 2007).

**Table 1:** Microbial species that induce Fe deficiency responses when applied to dicot plants.

Microbial species	Plant species	Mode appl.	Signals	Fe def. resp.	Fe genes	Fe Gr.	Refs
**Rhizobacteria**							
*Azospirillum brasilense*	*Solanum lycopersicum*	gm(a)	ET Auxin	Root hairs	nd	∧	Ribaudo et al., 2006
*Azospirillum brasilense*	*Cucumis sativus*	gm(ns)	nd	FCR, pH	*FIT FRO1 IRT1 HA1*	Fe	Pii et al., 2016b
*Bacillus subtilis*	*Arabidopsis thaliana*	gm(a)	VOCs	FCR, pH	*FIT FRO2 IRT1*	Fe	Zhang et al., 2009
*Arthrobacter agilis^∗^*	*Medicago truncatula*	gm(a)	VOCs	FCR, pH	nd	Fe ∧	Orozco-Mosqueda et al., 2013
*Pseudomonas simiae*	*Arabidopsis thaliana*	gm(a)	VOCs Auxin NO	FCR phenolics	*FIT FRO2 bHLH38 bHLH39 IRT1 F6′H1 MYB72 BGLU42 ABCG37*		Zamioudis et al., 2014, 2015; Stringlis et al., 2018b
*Enterobacter*^∗^ *Pseudomonas*	*Cucumis sativus*	gm(ns)	Auxin	FCR	nd	nd	Scagliola et al., 2016
*Paenibacillus polymyxa*	*Arabidopsis thaliana*	gm(a)	Auxin	FCR, pH, phenolics	*FIT FRO2 IRT1 MYB72 F6′H1*	Fe ∧	Zhou et al., 2016a
*Bacillus amyloliquefaciens*	*Arabidopsis thaliana*	gm(a)	VOCs Auxin NO	FCR	*FIT FRO2 IRT1*	Fe ∧	Wang J. et al., 2017; Zhou et al., 2017
*Burkholderia cepacia*	*Astragalus sinicus*	ri(s)	Auxin	FCR, pH, flavins	*FIT FRO2 IRT1 AHA2*	Fe ∧	Zhou et al., 2018
**Rhizofungi**							
*Trichoderma asperellum*	*Cucumis sativus*	gm(s)	nd	FCR	nd	Fe ∧	Zhao et al., 2014
*Trichoderma asperellum*, *T. harzianum*	*Arabidopsis thaliana*	gm(a)	VOCs	FCR, root hairs	*FIT FRO2 bHLH38 bHLH39 IRT1 MYB72*	nd	Martínez-Medina et al., 2017
*Trichoderma asperellum*, *T. harzianum*	*Solanum lycopersicum*	gm(a)	VOCs	FCR, root hairs	*FER FRO1 IRT1*	nd	Martínez-Medina et al., 2017

*Mode appl., mode of application of the microbes (gm, application to the growth medium; ri, root immersion; a, agar; ns, nutrient solution; s, soil); Signals, elicitors and hormones involved; Fe def. resp., Fe deficiency responses; FCR, ferric chelate reductase activity; pH, acidification; Fe genes, Fe acquisition genes; Fe Gr., increased shoot Fe concentration (Fe) and increased shoot growth (∧); nd, not determined; ^∗^, microbial species whose association with ISR is not yet clear.*

Over the last years, SAR and ISR have been extensively reviewed (Van Loon et al., 1998; Choudhary et al., 2007; Pieterse et al., 2012, 2014), so here we only discuss major principles in both responses. In systemic tissues, SAR is characterized by increased levels of the hormone salicylic acid (SA) which, through the redox-regulated protein NON-EXPRESSOR OF *PR* GENES1 (NPR1), activates the expression of a large set of *PATHOGENESIS-RELATED* (*PR*) genes, involved in defense responses (Figure 1; Pieterse et al., 1998, 2002, 2012, 2014; Van Loon et al., 1998; Choudhary et al., 2007). By contrast to SAR, ISR is generally mediated by an SA-independent pathway where JA and ET are the central players, and typically functions without *PR* gene activation (Figure 1; Pieterse et al., 1998, 2002, 2012, 2014; Van Loon et al., 1998; Choudhary et al., 2007). Despite these differences, it has been shown that NPR1 is also required for the JA/ET-dependent ISR triggered by rhizosphere microbes although its role seems to be different in both processes (Figure 1; Pieterse et al., 2014; Nie et al., 2017). In SA signaling, NPR1 is related to a function in the nucleus while in JA/ET signaling it is related to a cytosolic function (Pieterse et al., 2014). Despite these general differences, in some particular cases, ISR can require SA accumulation (Ryu et al., 2003; Alizadeh et al., 2013). Moreover, the signaling pathways involved in the induction of ISR can be different depending on the microbial species and the plant species (Ryu et al., 2003; Jankiewicz and Koltonowicz, 2012; Alizadeh et al., 2013).

The discovery of ISR occurred around 1991, when several researchers showed that root colonization by certain non-pathogenic bacterial races promoted the health of plants upon the stimulation of their defense responses (reviewed in Pieterse et al., 2014). After these pioneering works with bacteria, ISR was further extended to rhizosphere fungi, like *Trichoderma* spp. or *Piriformospora indica* (Table 1; Segarra et al., 2009; Alizadeh et al., 2013; Pieterse et al., 2014).

### How Is ISR Triggered by Beneficial Rhizosphere Microbes?

The ways beneficial rhizosphere microbes elicit ISR are not totally understood but several microbial elicitors have been proposed to be responsible for its onset. These elicitors, upon perception, would trigger the ISR through the action of diverse plant hormones (Figure 1; Pieterse et al., 2012, 2014; Sharifi and Ryu, 2018; Tyagi et al., 2018). Among these elicitors, there are Microbe-Associated Molecular Patterns (MAMPs) and other elicitors, like Volatile Organic Compounds (VOCs) or siderophores (Figure 1; Zhang et al., 2007, 2009; Jankiewicz and Koltonowicz, 2012; Orozco-Mosqueda et al., 2013; Pieterse et al., 2014; Zamioudis et al., 2015; Garnica-Vergara et al., 2016; Martínez-Medina et al., 2017; Sharifi and Ryu, 2018; Tyagi et al., 2018; Villena et al., 2018). MAMPs (when produced by pathogens are named Pathogen-Associated Molecular Patterns: PAMPs) are conserved microbial molecules released by the microbes, like flagellin, chitin, and lipopolysaccharides (LPS; Zeidler et al., 2004; Pieterse et al., 2014; Villena et al., 2018). VOCs are low molecular weight compounds derived from different biosynthetic pathways, with high vapor pressure and that can evaporate and disperse easily (Sharifi and Ryu, 2018; Tyagi et al., 2018). At present, over 1000 volatile compounds (including alkanes, alcohols, esters, ketones, sulfides, terpenoids, and sesquiterpenes) have been identified (Tyagi et al., 2018). Those derived from beneficial microbes can trigger drastic changes in plant growth patterns, generally by altering hormone signaling (Garnica-Vergara et al., 2016; Martínez-Medina et al., 2017; Sharifi and Ryu, 2018; Tyagi et al., 2018). Siderophores are Fe chelating agents released by the bacteria to further acquire Fe from the medium (Lemanceau et al., 2009; Aznar and Dellagi, 2015; Aznar et al., 2014, 2015).

Microbe-Associated Molecular Patterns are perceived by Pattern Recognition Receptors (PRRs) while other elicitors could be perceived by other Receptors (Rs), not known in all cases (Figure 1; Jankiewicz and Koltonowicz, 2012; Pieterse et al., 2014; Aznar and Dellagi, 2015; Aznar et al., 2015; Sharifi and Ryu, 2018; Tyagi et al., 2018; Villena et al., 2018). Upon perception, the elicitors trigger the ISR by affecting diverse plant hormones that act as central players in the plant immune signaling network leading to the activation of the defense responses (Figure 1; Pieterse et al., 2012, 2014; Sharifi and Ryu, 2018; Tyagi et al., 2018). In some cases, the microbes themselves can also produce different hormones, like auxin or cytokinins, that upon perception by the plant hormonal receptors (HRs) can cause changes in the root physiology and morphology (Grady et al., 2016; Scagliola et al., 2016; Asari et al., 2017; Kudoyarova et al., 2017; Patel and Saraf, 2017). Among the hormones implicated in the ISR, JA, ET, auxin, and NO play a key role (Knoester et al., 1999; Ton et al., 2001; Shoresh et al., 2005; Zhang et al., 2007; Van der Ent et al., 2008; Camehl et al., 2010; Acharya et al., 2011; Pieterse et al., 2014; Garnica-Vergara et al., 2016; Hossain et al., 2017; Martínez-Medina et al., 2017; Nie et al., 2017; Nascimento et al., 2018; Stringlis et al., 2018a).

### ISR Characteristics

One general characteristic of the microbial elicitors that induce ISR is their redundancy. This redundancy implies that microbial mutants defective in one elicitor can induce ISR through other elicitors (Meziane et al., 2005; Pieterse et al., 2014; Zamioudis et al., 2015). For example, the siderophore pseudobactin was as effective in inducing ISR as live bacteria but a mutant defective in pseudobactin biosynthesis was equally effective (Meziane et al., 2005). Beneficial ISR-eliciting microbes do not directly activate defense responses but sensitize the whole plant (a phenomenon called priming) for a faster and stronger activation of defense responses upon invasion by pathogens (Choudhary et al., 2007; Berendsen et al., 2012; Jung et al., 2012; Pieterse et al., 2014; Martinez-Medina et al., 2016). A high percentage of the genes, predominantly associated with defense responses, induced by the elicitors, like flagellin, are suppressed by the ISR-eliciting microbes to allow the establishment of a mutually beneficial interaction with the host root (Stringlis et al., 2018a). There is increasing evidence that beneficial soil-borne microbes hijack plant hormone signaling pathways to suppress the host defenses (Pieterse et al., 2012). This is also the case for the symbiotic relationship between legumes and rhizobia where the defense reactions set up by the plant are quickly suppressed, allowing microbial entry and the potential successful rhizobial establishment in plant roots (Guinel, 2015).

To elicit ISR, beneficial rhizobacteria must reach a minimal concentration equal to 10^5^–10^7^ colony forming unit (CFU) per gram of root for several days (Jankiewicz and Koltonowicz, 2012; Bakker et al., 2013; Pieterse et al., 2014). It should be noted that in the rhizosphere, the microbial density can range from 10^8^ to 10^9^ bacteria per gram and that its diversity is generally less than in the bulk soil since plant exudates specifically stimulate or repress members of the microbial community shaping the root microbiome (Figure 1; Berendsen et al., 2012; Bakker et al., 2013; Pii et al., 2016a; Stringlis et al., 2018b). In this sense, very recently it has been found that the release of the antimicrobial coumarin scopoletin by roots of Arabidopsis plants inoculated with the rhizobacterium *P. simiae* inhibits some soil-borne pathogens but not the rhizobacterium (Stringlis et al., 2018b). Coumarins are phenolic compounds that are also released by Fe-deficient roots to favor the Fe acquisition of plants (Schmid et al., 2014; Schmidt et al., 2014; Tsai and Schmidt, 2017a; Siwinska et al., 2018; Tsai et al., 2018; see also Section “Fe deficiency responses in dicot plants”). Consequently, the ISR-eliciting microbes, by inducing the release of coumarins and other Fe deficiency responses in plants, can improve the Fe nutrition of plants but, at the same time, they can benefit from a niche where their competitors are eliminated or restricted (Stringlis et al., 2018b).

## Interrelationship Between ISR and Fe Deficiency Responses in Dicot Plants

**FIGURE 2:**
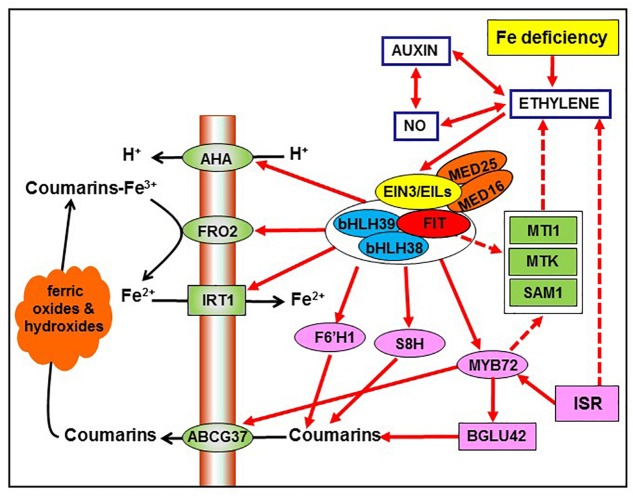
Possible interrelationship between ISR and Fe deficiency responses through the common participation of ethylene in both processes (see Figure 1). Ethylene, through the activation of the transcription factors FIT, bHLH38, and bHLH39, can up-regulate the expression of several genes associated with Fe deficiency, like *AHA*, *FRO2*, *IRT1*, *F6′H1*, and *S8H*. Additionally, FIT/bHLH38/bHLH39 can up-regulate the expression of *MYB72*, which activates the β-glucosidase BGLU42 and the phenolic efflux transporter ABCG37, both being implicated in the secretion of phenolic compounds, like coumarins. Moreover, FIT and MYB72 could indirectly act by affecting ethylene synthesis, through the upregulation of *SAM1*, *MTK*, and *MTI1*. Besides ethylene, auxin, and nitric oxide (NO) can also affect these Fe-related genes since they are closely interrelated with ethylene. Based on Lucena et al. (2015). For more details, see Section “Interrelationship between ISR and Fe deficiency responses.”

Since Fe acquisition is a limiting factor in most soils, Fe is a central player in the tripartite interaction among beneficial microbes, pathogens, and plants (López-Berges et al., 2013; Naranjo-Arcos and Bauer, 2016; Verbon et al., 2017). This close interrelation is in good agreement with the already described relationship between Fe homeostasis and defense responses against pathogens in plants (Lemanceau et al., 2009; Aznar et al., 2015; Verbon et al., 2017) and with the crosstalk between ISR and Fe deficiency responses (Pieterse et al., 2014; Verbon et al., 2017). The relationship between plant defense responses and Fe deficiency is complex and depends on several factors, like the plant genotype, the kind of pathogens and the intensity and duration of the deficiency. In some cases, plants are more tolerant to pathogens under conditions of Fe deficiency, probably because pathogens require an adequate quantity of Fe for full virulence (Kieu et al., 2012; López-Berges et al., 2013). However, in other cases, plants are more susceptible to pathogens under Fe-deficient conditions (Verbon et al., 2017 and references therein). The competition for Fe between soil-borne pathogens and their antagonistic microorganisms has been related to disease suppression; siderophores produced in the rhizosphere by PGPR can inhibit growth of the pathogens by depriving them of Fe (Verbon et al., 2017). In contrast to the negative effect of some soil-borne pathogens on Fe acquisition, there are several recent reviews showing an important role of beneficial rhizosphere microbes on the Fe nutrition of plants (Jin et al., 2014; Mimmo et al., 2014; Pii et al., 2015; İpek and Esitken, 2017). These microbes can directly improve Fe nutrition through the release of H^+^ and/or Fe- solubilizing compounds to soils, like siderophores and organic acids, or by inducing changes in root physiology and architecture, which can improve the acquisition of Fe and also of other nutrients (Orozco-Mosqueda et al., 2013; Jin et al., 2014; Mimmo et al., 2014; Zhao et al., 2014; Contreras-Cornejo et al., 2015; Pii et al., 2015, 2016b; Garnica-Vergara et al., 2016; Scagliola et al., 2016; Verbon and Liberman, 2016; Zhou et al., 2016a,b; Martínez-Medina et al., 2017; Sonbarse et al., 2017; Sharifi and Ryu, 2018; Stringlis et al., 2018a). In this way, it has been demonstrated that ISR-eliciting microbes can induce Fe deficiency responses in their host roots, such as enhanced ferric reductase activity, acidification of the rhizosphere, release of phenolics and flavins, and development of root hairs; and the expression of the genes associated with these responses, such as *FIT*, *bHLH38*, *bHLH39*, *MYB72*, *MYB10*, *FRO2*, *IRT1*, *AHA*, *F6′H1*, *BGLU42*, *ABCG37*, and others (Figure 1, 2 and Table 1; Ribaudo et al., 2006; Zhang et al., 2009; Zamioudis et al., 2014, 2015; Zhao et al., 2014; Pii et al., 2016b; Scagliola et al., 2016; Verbon and Liberman, 2016; Zhou et al., 2016a,b, 2018; Martínez-Medina et al., 2017; Verbon et al., 2017; see also Section “Fe deficiency responses in dicot plants” and Section “Rhizosphere microbial species that induce Fe deficiency responses and improve Fe acquisition”).

Since bacteria that elicit ISR can release siderophores to the medium, it has been speculated that perhaps these Fe chelating agents could deprive plants of Fe and in this way cause the induction of Fe deficiency responses (Van der Ent, 2008; Aznar et al., 2014; Pieterse et al., 2014; Aznar and Dellagi, 2015; Zamioudis et al., 2015). However, mutants defective in siderophore biosynthesis also induce Fe deficiency responses, which suggests that they could induce these responses through other mechanisms (Meziane et al., 2005; Pieterse et al., 2014; Zamioudis et al., 2015). A possibility could be through alteration of hormone biosynthesis and signaling in the plants. In this sense, the plant hormone ET has been implicated in both the activation of ISR (Knoester et al., 1999; Ton et al., 2001; Shoresh et al., 2005; Camehl et al., 2010; Pieterse et al., 2012; Garnica-Vergara et al., 2016; Hossain et al., 2017; Nie et al., 2017) and the activation of Fe deficiency responses (Figure 1, 2; reviewed in Lucena et al., 2015; Li and Lan, 2017; Romera et al., 2017). Besides ET, other hormones and signaling molecules, like auxin, and NO, have also been implicated in both processes (García et al., 2010; Acharya et al., 2011; Romera et al., 2011, 2017; Zamioudis, 2012; Jin et al., 2014; Contreras-Cornejo et al., 2015; Garnica-Vergara et al., 2016; Poupin et al., 2016; Stringlis et al., 2018a). Moreover, the root-specific MYB72 TF, that plays a key role in the onset of ISR (Van der Ent et al., 2008; Segarra et al., 2009; Zamioudis et al., 2014; Verbon et al., 2017; Stringlis et al., 2018b), is also essential for plant growth on low Fe conditions (Figure 2; García et al., 2011; Palmer et al., 2013; Zamioudis et al., 2014, 2015; Lucena et al., 2015; Verbon et al., 2017; see also Section “Fe deficiency responses in dicot plants”). This indicates that MYB72 is a node of convergence between ISR and Fe deficiency responses (Van der Ent et al., 2008; Segarra et al., 2009; Pieterse et al., 2014; Zamioudis et al., 2014, 2015; Verbon et al., 2017). All these results suggest that the regulatory pathways of ISR and Fe deficiency responses overlap. In the following paragraphs the main common components shared by ISR and Fe deficiency responses are described and analyzed.

### Ethylene and Other Hormones and Signaling Molecules

As previously stated (see Section “Induced Systemic Resistance”), ISR is generally mediated by a pathway where JA and ET are the central players (Figure 1; Pieterse et al., 1998, 2002, 2012, 2014; Van Loon et al., 1998; Choudhary et al., 2007). In this pathway, JA acts upstream of ET, and ET upstream of the NPR1 protein (Figure 1; Pieterse et al., 1998, 2002, 2012, 2014; Van Loon et al., 1998; Choudhary et al., 2007). NPR1 functions in the nucleus as a transcriptional coactivator of SA-responsive genes during the SAR pathway, but NPR1 also plays a cytosolic function in the JA/ET signaling during the ISR pathway (Figure 1; Pieterse et al., 2014). In relation to these components (ET, JA, SA, and NPR1), ET has been clearly implicated in the regulation of Fe deficiency responses in Strategy I plants and in rice, that presents characteristics of both Strategy I and II plant species (reviewed in Lucena et al., 2015; Li and Lan, 2017; Romera et al., 2017). However, the roles of the other components (JA, SA, and NPR1) on Fe deficiency responses are not yet clear. For instance, JA has been implicated in the regulation of Fe deficiency responses in Strategy I plants but as a suppressor of these responses (Maurer et al., 2011). In rice, JA has been shown to activate the expression of some Fe deficiency responses at the very early stages of the Fe deficiency (0.5–1 h) but strongly suppresses them later on (3–6 h; Kobayashi et al., 2016). In relation to the role of SA on the regulation of Fe deficiency responses, the results are contrasting. It was shown that Arabidopsis lines overexpressing the SA-inducible transcription factor OBF-BINDING PROTEIN 3 (OBP3) present upregulation of *bHLH038* and *bHLH039*, encoding two TFs that play a key role in the activation of Fe acquisition genes (Kang et al., 2003; see Section “Fe deficiency responses in dicot plants”). In the same way, exogenous application of SA to Arabidopsis plants upregulates the expression of *YELLOW STRIPE-LIKE1* (*YSL1*) and *YSL3*, which are involved in Fe translocation and homeostasis (Chen et al., 2014; Kumar et al., 2017). Shen et al. (2016) further found that Fe deficiency increases SA contents in shoots and roots of Arabidopsis plants, and that the SA biosynthesis defective mutant *phytoalexin deficient 4* (*pad4*) presents altered Fe deficiency responses, suggesting a link between SA and Fe deficiency. However, Maurer et al. (2014) found that SA and SA signaling through NPR1 (Figure 1) do not affect Fe deficiency responses. To complete this network of interactions, some Fe-related TFs, such as ILR3 (bHLH105; belonging to the IVc bHLH subgroup; Zhang et al., 2015; see also Section “Fe deficiency responses in dicot plants”), can affect JA and SA biosynthesis (Aparicio and Pallás, 2017). After analyzing all these results, it is clear that the role of JA and SA on the regulation of Fe deficiency responses in dicots deserves further research.

In relation to ET, this plant hormone is synthetized from the amino acid methionine, through a pathway requiring SAMS (S-adenosyl methionine synthetase), ACS [1-aminocyclopropane-1-carboxylic acid (ACC) synthase], and ACO (ACC oxidase; Sauter et al., 2013; Wang F. et al., 2013; Dubois et al., 2018):

$$\begin{array}{ccccccc} & \mathrm{SAMS} & & \mathrm{ACS} & & \mathrm{ACO} & \\ \mathrm{Methionine} & \to & \mathrm{SAM} & \to & \mathrm{ACC} & \to & \mathrm{Ethylene} \end{array}$$

Although ET’s mode of action is not fully understood, a linear signaling pathway has been proposed in Arabidopsis (Shakeel et al., 2013; Wang F. et al., 2013; Dubois et al., 2018):

ET−∥ET receptors→CTR1−∥EIN2→EIN3/EILs→ERFs→ET responses

In this signaling pathway, all the components are proteins and, within them, EIN3/EILs and ERFs are TFs (for more details, see Shakeel et al., 2013; Wang F. et al., 2013; Lucena et al., 2015; Dubois et al., 2018).

The participation of ET in the regulation of Fe deficiency responses was first proposed by Romera and Alcántara (1994) and has been further supported by many experimental data (recently reviewed in Lucena et al., 2015; Li and Lan, 2017; Romera et al., 2017). As more recent evidence, it should be mentioned that FIT (master regulator of Fe acquisition genes in Arabidopsis; see Section “Fe deficiency responses in dicot plants”) interacts with the EIN3 and EIL1 TFs, associated with ET signaling, and with MED16 and MED25, Mediators, to form a complex implicated in the transcription of Fe acquisition genes (Figure 2; Lingam et al., 2011; Yang et al., 2014; Brumbarova et al., 2015). In the same way, recently it has been found that the ERF4 and ERF72 TFs, that are also associated with ET signaling, are induced under Fe deficiency and participate in the regulation of Fe deficiency responses (Liu et al., 2017a,b). Fe deficiency causes the upregulation of genes involved in both ET synthesis, like *SAM*, *ACS*, *ACO*, *MTK*, *MTI1*, *MPK3*, and *MPK6*, and signaling, like *EIN2*, *EIN3*, *EIL1*, *EIL3*, *ERF4*, and *ERF72*, in roots of different dicot plants (García et al., 2010; Ye et al., 2015; Li and Lan, 2017; Romera et al., 2017). MTK (5-methylthioribose kinase) and MTI1 (5-methylthioribose-1-phosphate isomerase1) participate in the Yang cycle and are necessary for ET biosynthesis (Pommerrenig et al., 2011; Sauter et al., 2013). Both *MTK* and *MTI1*, besides their upregulation under Fe deficiency, are also regulated by ET, probably through FIT (Figure 2; Colangelo and Guerinot, 2004; García et al., 2010). *MTI1* (At2g05830) was previously identified as eIF-2B, a putative eukaryotic translation initiation factor (García et al., 2010; Pommerrenig et al., 2011). The mitogen-activated protein kinases 3 and 6 (MPK3/MPK6) are related to ET (Li et al., 2012; Contreras-Cornejo et al., 2015; Dubois et al., 2018) and can activate ACS2/6 in Fe deficiency-induced ET production (Ye et al., 2015).

Ethylene has been involved in the regulation of both morphological and physiological responses to Fe deficiency in Strategy I plants (Romera and Alcántara, 1994, 2004; Lucena et al., 2015; Li and Lan, 2017; Romera et al., 2017). In relation to the morphological responses, ET has been implicated in the formation of subapical root hairs, cluster roots, and transfer cells (reviewed in Lucena et al., 2015). In relation to the physiological responses, ET has been implicated in the upregulation of *FIT* (or its tomato homolog *FER*), *bHLH38*, *bHLH39*, and *MYB72*, encoding key TFs (Figure 2; Lucena et al., 2006; García et al., 2010; Lingam et al., 2011). bHLH38 and bHLH39 interact with FIT to form heterodimers that activate the expression of the Fe acquisition genes *FRO2* (ferric reductase) and *IRT1* (iron transporter) (Figure 2; Yuan et al., 2008; Wang N. et al., 2013). Similarly, the acidification capacity, depending on *AHA*-like genes (Colangelo and Guerinot, 2004), is also activated by FIT and consequently by ET (Figure 2; Waters et al., 2007; Lucena et al., 2015). In relation to the excretion of phenolics, it has been found that the expression of *F6′H1* and *S8H*, involved in their synthesis, is dependent on FIT (Figure 2; García et al., 2010; Schmid et al., 2014; Tsai and Schmidt, 2017a; Tsai et al., 2018; see also “Fe deficiency responses in dicots”). Consequently, both of them would also be dependent on ET (Figure 2). In supporting this view, *S8H* (At3g12900) is greatly induced in Fe deficient roots and drastically inhibited by ethylene inhibitors (García et al., 2010). Besides all the above genes related to Fe acquisition, ET also participates in the activation of *NAS1* (nicotianamine synthase1), *NAS2*, and *FRD3* (ferric reductase defective3), that are very important for internal Fe mobilization and homeostasis (García et al., 2010).

Besides its involvement in Fe deficiency responses, ET has also been implicated in the development of ISR triggered by root-colonizing microbes, acting downstream of JA (Figure 1; Knoester et al., 1999; Ton et al., 2001; Shoresh et al., 2005; Ribaudo et al., 2006; Van Loon et al., 2006; Van der Ent, 2008; Camehl et al., 2010; Pieterse et al., 2012; Zamioudis, 2012; Contreras-Cornejo et al., 2015; Garnica-Vergara et al., 2016; Hossain et al., 2017; Nie et al., 2017; Nascimento et al., 2018). In general, root colonization by ISR-eliciting microbes does not induce a direct enhancement of ET and JA biosynthesis (Knoester et al., 1999; Pieterse et al., 2014) except in some cases (Ribaudo et al., 2006; Contreras-Cornejo et al., 2015). However, the expression of genes involved in ET biosynthesis, like *ACSs* and *ACOs*, and signaling, like *ETR1*, *EIL3*, *CTR1*, and *ERFs*, is frequently upregulated in roots by ISR-eliciting microbes (Shoresh et al., 2005; Ribaudo et al., 2006; Van der Ent et al., 2008; Velivelli et al., 2015; Zamioudis et al., 2015; Poupin et al., 2016). For example, root colonization by *P. simiae* WCS417 induced the upregulation of *ACS2*, *ACS6*, and *EIL3* in Arabidopsis roots (Van der Ent et al., 2008; Zamioudis et al., 2015), which are also upregulated under Fe deficiency (García et al., 2010; Ye et al., 2015).

In both processes, ISR and Fe deficiency responses, ET can have a dual role. It is necessary for the activation of Fe deficiency responses (Lucena et al., 2015; Li and Lan, 2017; Romera et al., 2017) and for the onset of ISR (Knoester et al., 1999; Ton et al., 2001; Shoresh et al., 2005; Ribaudo et al., 2006; Pieterse et al., 2012, 2014; Garnica-Vergara et al., 2016; Hossain et al., 2017; Nie et al., 2017; Nascimento et al., 2018). However, when accumulated in excess, ET can have negative effects on the responses to Fe deficiency (Romera et al., 1999), on the growth of plants and on the mutualistic interactions with beneficial microbes (Pierik et al., 2007; Camehl et al., 2010; Gamalero and Glick, 2015; Nascimento et al., 2018). This dual role also occurs for the nodulation between legumes and rhizobia, where ET is crucial for the proper development of the rhizobial colonization but also acts as a negative regulator to limit the number of rhizobial infections (Zamioudis, 2012; Guinel, 2015). To avoid the detrimental effects of ET, some beneficial microbes and plant species possess the enzyme ACC deaminase, that eliminates the ET precursor ACC (Gamalero and Glick, 2015; Singh et al., 2015; Nascimento et al., 2018).

The participation of ET on morphological and physiological responses to Fe deficiency and on ISR probably follows different ET signaling pathways. While several Arabidopsis ethylene insensitive mutants, like *etr1*, *ein2*, *ein3*, and *eir1*, are blocked in their capacity to mount the ISR (Knoester et al., 1999; Ton et al., 2001; Van Loon et al., 2006; Camehl et al., 2010; Zamioudis, 2012; Alizadeh et al., 2013; Contreras-Cornejo et al., 2015; Hossain et al., 2017; Nie et al., 2017), and to develop morphological responses to Fe deficiency (Romera and Alcántara, 2004), they can induce most of the physiological responses to Fe deficiency (Romera and Alcántara, 2004; Lucena et al., 2006; García et al., 2010). These differences are perhaps related to the existence of an alternate route for ethylene signaling, besides the conventional one that includes EIN2 (see above; Shakeel et al., 2013). At this point, it has been suggested that for several physiological responses, ET could act through a pathway where EIN2 is not strictly required (Lucena et al., 2015).

Besides ET, other hormones and signaling molecules, such as auxin, GSH and NO have also been involved in both ISR and Fe deficiency responses (Ribaudo et al., 2006; Graziano and Lamattina, 2007; Bacaicoa et al., 2009, 2011; Chen et al., 2010; García et al., 2010, 2011, 2018; Acharya et al., 2011; Romera et al., 2011, 2017; Jin et al., 2014; Contreras-Cornejo et al., 2015; Shanmugam et al., 2015; Zamioudis et al., 2015; Garnica-Vergara et al., 2016; Poupin et al., 2016; Zhou et al., 2016a, 2017, 2018; Wang J. et al., 2017; Gullner et al., 2018; Kailasam et al., 2018; Sharifi and Ryu, 2018; Stringlis et al., 2018a; Sumayo et al., 2018; Tyagi et al., 2018). All of them increase in roots under Fe deficiency and frequently upon colonization of roots by ISR-eliciting microbes (Romera et al., 1999; Ribaudo et al., 2006; Graziano and Lamattina, 2007; Bacaicoa et al., 2009, 2011; Chen et al., 2010; Contreras-Cornejo et al., 2015; Shanmugam et al., 2015; Zamioudis et al., 2015; Zhou et al., 2016a, 2017; Wang J. et al., 2017; Kailasam et al., 2018). Some microbial elicitors, like VOCs or LPS, can affect ET, auxin or NO production and/or signaling, and in this way upregulate Fe-related genes (Zeidler et al., 2004; Zhang et al., 2007, 2009; Kwon et al., 2010; Liu and Zhang, 2015; Zamioudis et al., 2015; Garnica-Vergara et al., 2016; Zhou et al., 2017; Wang J. et al., 2017; Sharifi and Ryu, 2018; Tyagi et al., 2018). As examples, VOCs from *Bacillus subtilis* GB03 upregulated the expression of several ET biosynthesis genes (Kwon et al., 2010) and VOCs from *P. simiae* WCS417 or *Bacillus amyloliquefaciens* BF06 caused NO accumulation in Arabidopsis roots and upregulated several Fe-related genes (Zamioudis et al., 2015; Wang J. et al., 2017). Moreover, some of these hormones and signaling molecules can affect the perception of microbial elicitors. For example, the PRR for flagellin (FLAGELLIN-SENSING 2-FLS2) is regulated by ET (Boutrot et al., 2010).

Ethylene, auxin, and NO are closely interrelated since each one can affect the synthesis and/or action of the others (Figure 2; Ribaudo et al., 2006; Chen et al., 2010; García et al., 2011, 2018; Romera et al., 2011, 2017; Contreras-Cornejo et al., 2015; Garnica-Vergara et al., 2016; Poupin et al., 2016; Zhou et al., 2017). As a probe of their close interrelationship, *FIT*, *MYB72*, and other Fe- and ISR-related genes are similarly affected by ET, auxin, or NO treatments. They are upregulated by these molecules, or their precursors, and downregulated by their inhibitors (Graziano and Lamattina, 2007; Chen et al., 2010; García et al., 2010, 2011; Zamioudis et al., 2015; Wang J. et al., 2017; Zhou et al., 2017; Stringlis et al., 2018a; Sumayo et al., 2018).

In the last years, the roles of GSH and NO in the activation of responses to Fe deficiency are becoming more complex since several experimental results have shown that *S*-nitrosoglutathione (GSNO), derived from GSH and NO, specifically works in such an activation having a different role that the one of NO (García et al., 2018; Kailasam et al., 2018). NO, GSH, and GSNO have also been implicated in plant defense responses against pathogens, and NO and GSH in ISR (Zamioudis et al., 2015; Yun et al., 2016; Gullner et al., 2017, 2018). Moreover, in plant defense responses, NO and GSNO exhibit additive functions and, by extension, may have distinct or overlapping molecular targets (Yun et al., 2016). The roles of GSH and GSNO in these processes have been related to their capacity to detoxify toxins (by their conjugation with GSH), to their interconnection with reactive oxygen species and SA, and to their capacity to modulate the redox state of NPR1 and to *S*-nitrosylate defense-related TFs and transcriptional coregulators (Yun et al., 2016; Gullner et al., 2017, 2018). However, to our knowledge, only NO has been implicated in the signaling processes leading to the activation of Fe acquisition genes by ISR-eliciting microbes (Zamioudis et al., 2015) while GSH and GSNO have not yet been studied in relation to this activation, which deserves further research.

### MYB72 and Other Transcription Factors

Some years ago, it was found that the *MYB72* gene, encoding a TF, was greatly induced in Arabidopsis roots under Fe deficiency (Colangelo and Guerinot, 2004) and also upon their colonization with the ISR-eliciting rhizobacterium *P. simiae* WCS417 (Verhagen et al., 2004; Van der Ent, 2008; Van der Ent et al., 2008). Later on, *MYB72* upregulation has also been demonstrated with other ISR-eliciting microbes (Segarra et al., 2009; Alizadeh et al., 2013; Pieterse et al., 2014; Martínez-Medina et al., 2017; Verbon et al., 2017). Furthermore, Arabidopsis *myb72* knockout mutants are defective in the activation of ISR which suggests that MYB72 plays a key role in the early signaling steps of this process (Figure 2; Van der Ent, 2008; Van der Ent et al., 2008; Segarra et al., 2009; Alizadeh et al., 2013; Zamioudis et al., 2014, 2015; Stringlis et al., 2018b). However, overexpression of MYB72 did not result in enhanced resistance against any of the pathogens tested, demonstrating that MYB72 is not sufficient for the expression of ISR (Van der Ent et al., 2008). In both Fe deficiency and ISR, *MYB72* is upregulated along with *MYB10*, also encoding a TF (Colangelo and Guerinot, 2004; Zamioudis, 2012; Palmer et al., 2013; Zamioudis et al., 2015). MYB72 and MYB10 physically interact *in vivo* and function redundantly in regulating the expression of genes involved in the shikimate, the phenylpropanoid and the nicotianamine (NA) biosynthesis pathways (Zamioudis, 2012; Zamioudis et al., 2014, 2015; Stringlis et al., 2018b). Under Fe deficiency, both MYB72 and MYB10 act early in the Fe deficiency regulatory cascade to drive gene expression of *NAS2* and *NAS4*, two NA synthase genes (Palmer et al., 2013). An important difference of the participation of MYB72 and MYB10 in ISR and Fe deficiency responses is that the Arabidopsis *myb72* mutants are defective in the activation of ISR (see above) while they behave apparently normal when grown on alkaline soil, a condition that favors Fe deficiency (Palmer et al., 2013). However, the *myb10myb72* double mutant displays seedling lethality when grown on alkaline soil (Palmer et al., 2013). This suggests that MYB10 and MYB72 have overlapping roles in relation to Fe deficiency (Palmer et al., 2013). In relation to ISR, MYB72, and MYB10 coordinately suppress the expression of a large group of defense-related genes upon root colonization by *P. simiae* WCS417, enabling the bacteria to establish long-term associations with host roots (Zamioudis, 2012). The bacteria can also colonize roots of the *myb72* mutant (Van der Ent et al., 2008), suggesting that MYB10 may compensate in defense suppression (Zamioudis, 2012).

MYB72 is consequently a node of convergence between ISR and Fe deficiency responses in dicots. This convergence is further supported when considering that *MYB72* expression is controlled by FIT either under Fe deficiency or upon colonization of roots by ISR-eliciting microbes (Figure 2; Colangelo and Guerinot, 2004; Sivitz et al., 2012; Palmer et al., 2013; Zamioudis et al., 2015). Moreover, FIT interacts with the bHLH38 TF to control *MYB72* expression upon colonization of roots by ISR-eliciting microbes (Zamioudis et al., 2015), as occurred with *FRO2* and *IRT1* expression under Fe deficiency (Yuan et al., 2008). *FIT*, *bHLH38*, and *MYB72* expression is activated by ET (García et al., 2010, 2011), which suggests a connection between the regulation of Fe deficiency responses and ISR through this plant hormone. In supporting this view, several *P. simiae* WCS417-inducible MYB72 target genes, like *BGLU42* (β-glucosidase), *CYP71B5* (cytochrome P450), *At5g55620* (unknown function), and *bHLH39* (TF), are all induced by Fe deficiency and also activated by ET (García et al., 2010; Zamioudis et al., 2014). Moreover, *MYB72* and *BGLU4*2 present ET-responsive elements in their promoters (García et al., 2010). Similarly, there are genes associated with the biosynthesis and release of coumarins to the rhizosphere, like *F6′H1*, *S8H*, and *ABCG37*, that are upregulated both under Fe deficiency and upon colonization of roots by ISR-eliciting microbes. These genes are dependent on FIT (*F6′H1*, *S8H*) or MYB72 (*ABCG37*) and, consequently, on ET (Figure 2; García et al., 2010; Schmid et al., 2014; Schmidt et al., 2014; Zamioudis et al., 2014; Tsai and Schmidt, 2017a; Siwinska et al., 2018; Tsai et al., 2018; see also Section “Fe deficiency responses in dicot plants”).

The results about the relationship of MYB72 and ET are controversial. For example, Van der Ent et al. (2006) showed that *MYB72* transcript levels accumulated after treatment with the ET precursor ACC and that they did not accumulate in the Arabidopsis ethylene insensitive mutant *ein2-1* upon root colonization with the ISR-eliciting bacterium *P. simiae* WCS417. However, later on, these authors found that *MYB72* transcript levels did not accumulate after treatment with ACC while they accumulated in the *ein2-1* mutant upon treatment with *P. simiae* WCS417 (Van der Ent et al., 2008). After these latter results, they concluded that *MYB72* expression was not regulated by ET (Van der Ent et al., 2008). Similarly, it was found, by using yeast two-hybrid screening, that MYB72 physically interacted *in vitro* with EIL3, a TF associated with ET signaling (Van der Ent et al., 2008) while later on it was found, by using Bimolecular Fluorescence Complementation, that MYB72 and EIL3 did not interact *in vivo* (Zamioudis, 2012). Curiously, *EIL3* expression is upregulated both under Fe deficiency (García et al., 2010) and upon colonization of roots with *P. simiae* WCS417 (Van der Ent et al., 2008). Today, there is enough evidence to support the regulation of *FIT*, *bHLH38*, and *bHLH39* by ET and, consequently, the one of *MYB72*. For example, both *FIT* and *MYB72* are upregulated by ET treatment (García et al., 2010). In relation to FIT regulation, it has been shown that the ET-signaling TFs EIN3 and EIL1 interact with FIT to favor its stability and activity (Lingam et al., 2011; Yang et al., 2014; Brumbarova et al., 2015; see Subsection “Ethylene and other hormones and signaling molecules”). The upregulation of *MYB72* in the *ein2-1* mutant could be explained by taking into account the upregulation of *FIT*, *bHLH38*, and *bHLH39* in this mutant (García et al., 2010). This could be related to the existence of an alternate route for ethylene signaling, besides the conventional one including EIN2 (Shakeel et al., 2013; Lucena et al., 2015). The complexity of the relationship between MYB72 and ET is also manifested when considering that MYB72 can also affect ET biosynthesis: the *SAM1* gene, encoding a SAM synthetase enzyme involved in ET synthesis, is upregulated by MYB72 (Figure 2; Zamioudis et al., 2014) and also is upregulated under Fe deficiency in Arabidopsis roots (García et al., 2010). In accordance with the influence of MYB72 on ET, the exogenous application of ACC induced wild-type levels of resistance in the Arabidopsis *myb72-1* mutant, suggesting that MYB72 acts upstream of ET in the ISR pathway (Van der Ent et al., 2008).

Besides the participation of the MYB72, MYB10, FIT, bHLH38, and bHLH39 TFs in both ISR and Fe deficiency responses, there are other TFs that also play a key role in both processes. Among them, the EIN3/EIL1 (related to ET signaling) and MED16 (Mediator) TFs can be highlighted (see Subsection “Ethylene and other hormones and signaling molecules”). These TFs are required to activate the expression of Fe acquisition genes by interacting with FIT (Figure 2; Lingam et al., 2011; Yang et al., 2014; Zhang et al., 2014; Brumbarova et al., 2015; Lucena et al., 2015). In relation to ISR, the Arabidopsis *ein3-1* mutant did not express ISR in response to treatment with the bacterium *P. simiae* WCS417, which suggests that the EIN3 TF also plays a key role in this process (Knoester et al., 1999). MED16 is a key component in the JA/ET-mediated immunity against necrotrophic pathogens (Wang et al., 2015). In addition, and besides affecting the expression of Fe acquisition genes, it greatly influences the expression of *MYB72* and *MYB10* (Zhang et al., 2014), two important components in both Fe deficiency responses and ISR (see above). Probably, the physical interaction of MED16 with FIT is necessary for the activation of *MYB72* and *MYB10* expression (Figure 2).

### Internal Fe Content

Eethylene, auxin, and NO greatly activate the expression of Fe acquisition genes in plants grown with low levels of Fe (or without Fe), but have much less effect in plants grown with high levels of Fe (Lucena et al., 2006; Graziano and Lamattina, 2007; Chen et al., 2010; García et al., 2011). This suggests that the upregulation of Fe acquisition genes does not solely depend on hormones and signaling molecules (such as ET, auxin, or NO), that would act as activators, but also on the internal Fe content of plants, that would act as a repressor (Lucena et al., 2006; García et al., 2011, 2013, 2018; Romera et al., 2011, 2017). However, different results suggest that total Fe in roots is not the repressor of Fe acquisition genes but instead it is a Fe-related signal moving from shoots to roots through the phloem (García et al., 2013, 2018). Very recently, it has been found that this shoot Fe-related signal can affect the synthesis of ET on roots (García et al., 2018). To integrate all these regulatory components, a model has been proposed where ET/auxin/NO act as activators of Fe acquisition genes while a phloem Fe-related signal acts as repressor (García et al., 2011, 2013, 2018; Lucena et al., 2015; Romera et al., 2017).

In relation to ISR, there are also several results showing that the effects of ISR-eliciting microbes on the induction of Fe deficiency responses could also be dependent on the Fe concentration of the medium. For example, the expression of two Fe-related genes, like *MYB72*, and *FRO2*, was induced by *P. simiae* WCS417 independently of the Fe concentration in the medium but their absolute values were decreased when the Fe concentration increased (Zamioudis et al., 2015). Other examples, the expression of *FIT*, *IRT1* and *FRO2*, and the ferric reductase activity, decreased in Arabidopsis plants inoculated with *P. polymyxa* BFKC01 when the Fe concentration in the medium increased (Zhou et al., 2016a). The expression of *FIT*, *IRT1*, *FRO1*, and *HA1*, and the ferric reductase activity, in cucumber plants inoculated with *Azospirillum brasilense* Cd(DSM-1843) or other rhizobacterial species, decreased when the Fe concentration in the medium increased (Pii et al., 2016b; Scagliola et al., 2016). The acidification capacity induced by the rhizobacterium *Arthrobacter agilis* in *Medicago truncatula* roots was lower in plants grown with high levels of Fe than in those grown with low levels of Fe (Orozco-Mosqueda et al., 2013). Moreover, the expression of several Fe-related genes induced by *P. simiae* WCS417, like *MYB72*, *FRO2*, and *IRT1*, is transitory (Zamioudis et al., 2015). This suggests that, after the induction of the Fe acquisition genes by ISR-eliciting microbes, plants acquire enough Fe and turn off these genes, to avoid toxicity and to conserve energy. The same occurs when plants induce the Fe acquisition genes under Fe deficiency and, as a consequence, they get sufficient Fe (Vert et al., 2003; Lucena et al., 2015).

## Rhizosphere Microbial Species That Induce Fe Deficiency Responses and Improve Fe Acquisition

As previously stated, beneficial rhizosphere microbes can contribute to improve Fe acquisition. This is most likely due to their capacity to induce Fe deficiency responses, such as enhanced ferric reductase activity, acidification of the rhizosphere, release of phenolics and flavins, and development of root hairs (see Section “Interrelationship between ISR and Fe deficiency responses in dicot plants”). These Fe deficiency responses are induced in a similar way as they are induced under Fe deficiency conditions. For example, ISR-eliciting microbes induce the upregulation of the genes associated with the Fe deficiency responses, like *FRO2*, *IRT1*, *AHA*, *F6′H1*, *BGLU42*, *ABCG37*, and others, and these genes are activated by the TFs that activate them under Fe deficiency, like FIT(FER), bHLH38, bHLH39, MYB72, and MYB10 (Figure 2; Zhang et al., 2009; Palmer et al., 2013; Zamioudis et al., 2014, 2015; Zhao et al., 2014; Pii et al., 2016b; Scagliola et al., 2016; Verbon and Liberman, 2016; Zhou et al., 2016a, 2017, 2018; Martínez-Medina et al., 2017; Verbon et al., 2017; Wang J. et al., 2017; Stringlis et al., 2018a,b). Moreover, the hormones and signaling molecules related to the activation of these TFs, like ET, auxin, and NO, are similar in both ISR and Fe deficiency responses (see Subsection “Ethylene and other hormones and signaling molecules”).

Among the beneficial rhizosphere microbes that can activate the ISR are rhizobacteria, like *P. simiae* (syn. *P. fluorescens*), *B. subtilis*, *P. polymyxa* and *A. brasilense*; rhizofungi, like *Trichoderma* spp.; mycorrhizal fungi, like *Rhizophagus irregularis* (syn. *Glomus intraradices*) and *P. indica*; and non-pathogenic races of *Fusarium oxysporum* (Segarra et al., 2009; Zhang et al., 2009; Patil et al., 2011; Pieterse et al., 2012, 2014; Alizadeh et al., 2013; Zamioudis et al., 2014, 2015; Zhao et al., 2014; Pii et al., 2016b; Martínez-Medina et al., 2017; Verbon et al., 2017). In the case of mycorrhizal fungi, the enhanced defensive capacity provoked by them is also named MIR (“Mycorrhiza-Induced Resistance”) and can favor P acquisition (Jung et al., 2012; Cameron et al., 2013). Furthermore, it has been suggested that MIR can involve an ISR component elicited by bacteria in the mycorrhizosphere (Cameron et al., 2013). This is supported by the synergistic effects in defense responses when both arbuscular mycorrhiza and rhizobacteria are simultaneously applied (Pérez de Luque et al., 2017). The synergy between both kind of microbes paves the way to study the consortia of mycorrhiza and rhizobacteria in relation to the acquisition of Fe and P, and perhaps of other nutrients.

In Table 1 are summarized several rhizobacteria and rhizofungi species (most of them trigger ISR) that have been shown to induce Fe deficiency responses and to improve Fe acquisition and/or growth when applied to dicot plants. In Table 2 are summarized the ones that have been shown to cause similar effects when applied to dicot plants grown in calcareous soils (or in artificial calcareous soils).

**Table 2:** Microbial species that improve Fe nutrition when applied to dicot plants grown in calcareous soils (or in artificial calcareous soils).

Microbial species	Plant species	Mode appl.	Fe def. resp.	Fe Gr.	Refs
**Rhizobacteria**					
*Bacillus subtilis*	*Manihot esculenta*	ri	nd	Fe ∧	Freitas et al., 2015
*Paenibacillus polymyxa*	*Arabidopsis thaliana*	ri	nd	Fe	Zhou et al., 2016a
*Bacillus* sp. *Agrobacterium* sp.^∗^ *Alcaligenes* sp.^∗^ *Pantoea* sp.^∗^	*Pyrus communis*	ri	FCR organic acids	Fe	Ipek et al., 2017
*Alcaligenes* sp.^∗^ *Pantoea* sp.^∗^	*Malus domestica*	i	FCR organic acids	Fe	Aras et al., 2018
*Bacillus* sp. *Agrobacterium* sp.^∗^ *Alcaligenes* sp.^∗^ *Staphylococcus* sp.^∗^	*Prunus persica*	ri	FCR organic acids	Fe	Arıkan et al., 2018
**Rhizofungi**					
*Trichoderma asperellum*	*Lupinus albus*	gm and ri	nd	Fe	de Santiago et al., 2009
*Trichoderma asperellum*	*Cucumis sativus*	ri	nd	Fe	de Santiago et al., 2013

*Mode appl., mode of application of the microbes (gm, application to the growth medium; i, through the irrigation system; ri, root immersion). FCR, ferric chelate reductase activity; Fe Gr., increased shoot Fe concentration (Fe) and increased shoot growth (∧); nd, not determined; ^∗^, microbial species whose association with ISR is not yet clear.*

## Concluding Remarks and Future Perspectives

The ability of ISR-eliciting microbes to trigger both defense responses and Fe deficiency responses opens the way to use them as biopesticides and also as Fe biofertilizers. This represents a very important opportunity to diminish the application of fertilizers and pesticides in a more sustainable agriculture. However, and in relation to Fe nutrition, the use of ISR-eliciting microbes is in its infancy since it is not sufficiently known the behavior of these microbes on crops grown in calcareous soils. Most research works about the realtionship of these microbes with the Fe nutrition of plants have been carried out with Arabidopsis plants grown on agar plates. For their application to crop plants in the field it would be also necessary to study the behavior of these microbes with plant species growing in calcareous soils, including their capacity to thrive in these soils and to compite with wild soil microbes. More research is also needed to know the best ways for their application, by analyzing and comparing, both biologically and economically, the different possibilities, like direct application to soil, root immersion of plantlets before transplanting them (in the case of crop trees), application to seeds, and application into the irrigation systems (probably as spores). In the same way, it is necessary to study whether it is better to apply individual microbial species or consortia of different microbial species (Alizadeh et al., 2013; Sonbarse et al., 2017). In this latter case, and since ET can play a dual role in both ISR and Fe deficiency responses, it would be interesting to analyze the interactions between plant species and microbial species possesing the ACC deaminase enzyme and those that do not. Anyway, the research about ISR-eliciting microbes and Fe nutrition is a very fascinating topic for the near future.

## Author’s Note

We apologize to authors whose works were not cited in this review due to our ignorance and to manuscript length restrictions. We encourage authors with papers relating ISR and Fe nutrition in dicot plants to send them to us: we are preparing a website (http://www.uco.es/rhizosferrum) to keep updated all the microbial species that elicit ISR, induce Fe deficiency responses, and improve Fe acquisition.

## Author Contributions

FR, MG, and CL revised the information related to ISR and Fe deficiency signaling. RP-V revised the information related to volatile compounds. EA, JR, MAA, and MA revised the information related to the different microbial species and their effects on Fe nutrition. FR wrote a first draft of the manuscript. JR, RP-V, and AM-M corrected and improved the manuscript.

## Conflict of Interest Statement

The authors declare that the research was conducted in the absence of any commercial or financial relationships that could be construed as a potential conflict of interest.
